# Presence of CRISPR CAS-Like Sequences as a Proposed Mechanism for Horizontal Genetic Exchanges between Trichomonas vaginalis and Its Associated Virus: A Comparative Genomic Analysis with the First Report of a Putative CRISPR CAS Structures in Eukaryotic Cells

**DOI:** 10.1155/2023/8069559

**Published:** 2023-11-27

**Authors:** Azra Kenarkoohi, Amir Abdoli, Arman Rostamzad, Mahmoud Rashnavadi, Razi Naserifar, Jahangir Abdi, Morteza Shams, Arezoo Bozorgomid, Sepideh Saeb, Dhurgham Al-Fahad, Kosar Khezri, Shahab Falahi

**Affiliations:** ^1^Department of Laboratory Sciences, School of Allied Medical Sciences, Iran; ^2^Department of Microbiology, Faculty of Medicine, Ilam University of Medical Sciences, Ilam, Iran; ^3^Zoonotic Diseases Research Center, Ilam University of Medical Sciences, Ilam, Iran; ^4^Zoonoses Research Centre, Jahrom University of Medical Sciences, Jahrom, Iran; ^5^Department of Biology, Faculty of Sciences, Ilam University, Ilam, Iran; ^6^School of Veterinary Medicine, Ilam University, Ilam, Iran; ^7^Infectious Diseases Research Center, Health Institute, Kermanshah University of Medical Sciences, Kermanshah, Iran; ^8^Qaen School of Nursing and Midwifery, Birjand University of Medical Sciences, Birjand, Iran; ^9^Pharmaceutical Department, College of Pharmacy, University of Thi-Qar, Iraq

## Abstract

**Introduction:**

Trichomonas vaginalis genome is among the largest genome size and coding capacities. Combinations of gene duplications, transposon, repeated sequences, and lateral gene transfers (LGTs) have contributed to the unexpected large genomic size and diversity. This study is aimed at investigating genomic exchange and seeking for presence of the CRISPR CAS system as one of the possible mechanisms for some level of genetic exchange. *Material and Methods*. In this comparative analysis, 398 publicly available Trichomonas vaginalis complete genomes were investigated for the presence of CRISPR CAS. Spacer sequences were also analyzed for their origin using BLAST.

**Results:**

We identified a CRISPR CAS (Cas3). CRISPR spacers are highly similar to transposable genetic elements such as viruses of protozoan parasites, especially megavirals, some transposons, and, interestingly, papillomavirus and HIV-1 in a few cases. *Discussion*. There is a striking similarity between the prokaryotes/Archaean CRISPR and what we find as eukaryotic CRISPR. About 5-10% of the 398 T. vaginalis possess a CRISPR structure.

**Conclusion:**

According to sequences and their organization, we assume that these repeated sequences and spacer, along with their mentioned features, could be the eukaryotic homolog of prokaryotes and Archaean CRISPR systems and may involve in a process similar to the CRISPR function.

## 1. Introduction

Trichomonas vaginalis (T. vaginalis) is the causative etiology of the most prevalent nonviral sexually transmitted infection in humans called trichomoniasis and was first introduced in 1836. This protozoan is a unicellular [1, 2], haploid member of parabasalids that infect a wide range of animals and humans, wildlife, livestock, and pets [3, 4]. In 2007, a whole genome sequence project of T. vaginalis was performed and showed an interesting big genome with 46,000 to 60,000 genes encoded on six chromosomes, which is among the largest genome size and coding capacities ever seen [1, 5]. Repeated sequences and transposable elements form up to 45% of the genome. Combinations of gene duplications, transposon, repeated sequences, and LGTs (lateral gene transfers) have contributed to the unexpectedly large genomic size (~170 Mbp) [5–7]. The high amount of lateral gene transfer and duplication events are responsible for the large genomic size in this organism that led to the expansion of diverse families of genes. This parasite is dependent on the mucosa and associated with the microbiota of the vagina, but interactions between the T. vaginalis parasite and the resident bacteria in the vaginal area are more complex and clearly unknown phenomena [6]. T. vaginalis express a large number of functional noncoding genes as transcript-only copies in nonprotein-coding gene regions of the organism genome [5, 7].

Despite a very long history of efforts and many struggles, as well as extensive research into the development of more diverse and effective drugs and vaccines, infectious diseases still occupy a large part of human concerns in the field of health and longevity [8, 9]. Among them, parasitic diseases have gained particular importance due to their unknown aspects and their emerging-remerging nature [10–14].

The cell and molecular aspects of the reciprocal interaction between parasite and vaginal microbiota are not clearly elucidated. Lateral genomic-transferred genes are stably integrated and maintained and act as the way that helps genetic information can transfer from one to unrelated genomes and cells. It seems that LGTs are an indispensable mechanism in genome evolution and diversity. LGTs, by transmitting infection-related genetic elements and natural selection factors between unrelated cells, may have critical importance in the evolution of an organism's lifestyle [15]. Infectious agents use various mechanisms to overcome cell defence systems and host factors, the establishment of infection, resistance to chemotherapy and antibiotics, and induction of various symptoms and complications in the host, many of which are still unknown to us, or we have little information about them [3, 8, 16, 17]. Despite numerous studies, many ambiguities in our knowledge about the most basic cellular and molecular processes of T. vaginalis remain. It is approved that genetic exchange occurs between microbes. This theory is particularly important for its implications in rising resistance to metronidazole or parasite virulence. Several comparative genomic analysis studies have shown the possibility of genetic exchange during the parasite's life cycle [18]. Complete genome analyses showed significant gene acquisition from prokaryotes by eukaryotic cells using the LGT process [6]. Whole genome analysis and walking on T. vaginalis can lead to understanding the core biological mechanisms regarding genome expansion, gene duplication, and parasite-host interaction [1, 18].

About 70% of the Trichomonas genome is composed of repeated sequences and transposon elements, which reflects a massive, evolutionarily expansion of the genome that part of these sequences are members of the high copy number genes cluster, and 40,000 transposable element (TE) genes are highly similar and distributed among 59 TE families [1, 3, 6, 19]. Another isolate, Trichomonas foetus strain K, has a highly repetitive and abundant genome [3]. The genome's structure, organization, and size in T. vaginalis and the results of several analyses proposed that T. vaginalis may participate in gene exchange as early as this or in its recent evolutionary history [4]. The reason why T. vaginalis have such a large and unique structure genome and how the gene expansion process occurred is unclear [19].

The first description of giant viruses in amoeba revolutionized the scientific viewpoint on virus research and genomics, leading to specific challenges in genetic diversity and evolutionary biology. Mimivirus was isolated by coculturing with Acanthamoeba spp. and was the most giant known virus [20, 21].

Several new or putative virus families and groups have been described to date. These families are Mimiviruses, Marseilleviruses, Pandoraviridae, faustoviruses from Asfarviridae, Totiviridae, Pithovirus, Mollivirus, and virophages. Also, some of these viruses may form new giant virus families. These groups of viruses are phylogenetically related to other large DNA virus families proposed to be classified in Megavirales as a new order. Mimiviruses and Marseilleviruses detected in human specimens and probably linked to some pathogenic issues and other members appear to be distributed in different environments [21, 22]. T. vaginalis infected by double-stranded RNA viruses from the Totiviridae family called the genus Trichomonasvirus. The virus's exact role in the organism's virulence remains unknown [17, 18]. Repeat families of T. vaginalis genomes can belong to virus-like sequences, transposable sequences, retrotransposon family, and unclassified sequences [1, 3, 5, 6, 15, 17–19, 23, 24]. The absence of a precise correlation between the average pairwise difference between nucleotide sequences and copy number of genes suggests a sudden expansion as a mechanism for acquisition of these repeats [19, 20, 25]. Amoebae can serve as a “melting pot” for genetic exchange, where microorganisms in amoeba trade their genetic content [6, 17, 22, 23, 26]. Furthermore, being a place of exchange, the amoeba can participate in genetic exchanges. It is shown that amoebae can transmit some genes to some viruses by unknown mechanisms [26, 27]. The term CRISPR represents clustered regularly interspaced short palindromic repeat, which was initially reported in the Escherichia coli genome and, a few years later, the Archaea domain. Similarities and homology were also discovered between CRISPR's spacer regions and sequences of bacteriophages and plasmids that infect bacteria and Archaean. This discovery led to an understanding of CRISPR's function in the adaptive immune system in prokaryotic life [28, 29]. The CRISPR repeats typically vary in size from 23 to 47 nucleotides, which are transcribed and cleaved into mature CRISPR RNAs [30]. A well-accepted theory in evolutionary biology is that CRISPR can be found only in most Archaea, about 40% of prokaryotic cells, and a few prokaryotic viruses (bacteriophages) [29, 31]. Microbial CRISPR CAS systems known so far are involved in pathogenesis and virulence, drug resistance, genetic processes, and defence against the attack of foreign microorganisms or genetic elements and host cell defence system [8, 32–39]. The exact mechanism of genetic exchange between different organisms and amoeba is still not fully understood. Also, different mechanisms may play a role in this exchange due to the diversity of organisms involved. Here, we report the probability for functional occurrence of these systems in eukaryotic genomes naturally and the introduction of CRISPR CAS as one of the mechanisms that play an important role in genetic exchange between Trichomonas and giant viruses.

## 2. Material and Methods

### 2.1. Sequences and Data Set

This comparative analysis investigated 398 publicly available Trichomonas vaginalis complete genomes or whole genome shotgun sequencing projects from GenBank of different genotypes. Information about downloaded genomes was obtained in the GenBank (http://www.ncbi.nlm.nih.gov/genome/) database, the keyword “Trichomonas vaginalis whole genome” or “Trichomonas vaginalis complete genome” was used, for retrieved sequences of one isolate of Trichomonas vaginalis whole genome selected to explore by clicking “See Genome Assembly and Annotation report”.

Three hundred ninety-eight isolates were downloaded and saved as FASTA format for seeking CRISPR CAS cluster in the following steps. After analyzing the presence of CRISPR CAS sequences by CRISPRCasFinder and assessing the repeat sequences, 100 spacer sequences from CRISPR detected in Trichomonas vaginalis strains obtained by evidence level 3 or 4 were also analyzed for their origin using Basic Local Alignment Search Tool (BLAST). Exploring Trichomonas vaginalis using a comparative full genome analysis approach has advantages and drawbacks. Assessing the full genome (or chromosome) sequences detailed information about genome length and organization, revealing critical data about genome composition and diversity and how genome segments are shared and exchanged through the entire genome. However, the lack of full genome sequence for most amoeba is one of the main limitations in using genomic data to analyze gene exchange.

## 3. Characterization of CRISPR Cas Systems

In our survey, CRISPR was found using CRISPRCasFinder (https://crisprcas.i2bc.paris-saclay.fr/CrisprCasFinder/Index) with predefined and manual proofreading of the parameter.

The CRISPRCasFinder is usually used for easy, fast, and reliable detection of CRISPRs and cas genes in the sequence data submitted in different form by the user and is an update of the CRISPRFinder software with more sensitivity and higher specificity and indication on the CRISPR orientation. Identification of cas genes in this program was done using MacSyFinder to determine CRISPR Cas type and subtype. Also, for more investigation and searching for possible Cas homolog in the genome, a search for identification of Cas or its homolog was performed on the Pfam and TIGRFAMs databases using the BLAST program. BLAST can be used as a reliable tool to infer similarity and functional and evolutionary connections between two genomes and sequences or to identify members of gene families. Searches for similarities between the spacer and possible ancestor were performed using the NCBI nucleotide BLAST service at http://www.ncbi.nlm.nih.gov/BLAST/ against a complete database or limited database like databases from RefSeq of virus sequences (date 2019-06-16) or on the database like Env-nt (date 2019-06-16) at NCBI. The default parameters of the program were used. The BLAST tool detects sequences of local similarity between genomes. This program searches the protein or nucleotide sequences and compares them to different databases and presents the results based on statistical significance between matches [40, 41]. Due to the short size of the spacer sequences (<50 nt) as well as the large size of data in main databases, the significance and accuracy of the BLAST performance were again confirmed by an iterative process. First, only similar sequences with the best Expect-values (E-values) were selected. The E-value cutoff was set because of unexpected discontinuity between related and unrelated sequences to another similar sequences. Finally, sequence similarity, E-value, and sequence relationship were used as search criteria for each spacer to confirm positive results and recognize false negatives [40]. CRISPRCasFinder uses several criteria for selecting sequences as true repeats and spacers, and the percentage of identity between spacers must be below 60%. The presence of related proteins in the vicinity of the cluster and its folding properties is a more decisive criterion that would override such homology thresholds [42].

## 4. Results

### 4.1. Presence of CRISPR System in Trichomonas vaginalis

**Figure 1:**
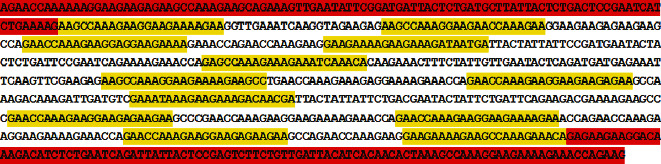
A representative CRISPR in Trichomonas vaginalis genome: 24 repeats (yellow highlight) and 23 spacers and leader sequence (red highlight).

We identified an unusual long chain of 20-60 bp tandemly repeated sequences, interspaced by 15-55 bp unique spacer sequences. Repeated sequences also possess short inverted repeats, like what Mojica et al. reported in the Archaea [29, 43]. These repeated sequences, in conjunction with spacers, extend for about 600 to 1500 bp and can naturally lead to the characteristic configuration of secondary structures as described for prokaryotic CRISPRs. Repeated sequences and spacers are flanked by up to 100 bp unique sequences. Repeats and spacer organization detected in our survey are shown (Figure 1).

All the loci reveal the same infrastructure, such as the repeated sequences of a conserved 20-60 bp and a spacer (12–55 bp). The size of the regions, as determined by comparative analysis, directly relates to the number of repeats and spacers in a given row [44]. In our survey, proposed CRISPR sequences were observed in different evidence levels between 1 and 4. These levels are assigned based on overall length, minimal and maximal repeats and spacer length, maximal and minimal spacer size in relation to repeat size, the highest percentage of identity between spacers allowed, the number of mismatches allowed between repeats and number, and similarity to consensus sequence and are widespread in the genomes of examined organisms. But based on CRISPRCasFinder and other evidence level criteria, we only accept sequences with evidence level 4 as real CRISPR; we found the eukaryotic CRISPR (eCRISPR) in approximately 1-5 percent of assessed genome (more than 50000 sequences) of different family, during the survey, so far. All reported eCRISPR were related based on sequence identity and may be functional to bacterial and archaeal CRISPR. Comparative analysis showed the common characteristics of the eCRISPR and pCRISPRs, as they are perched in intergenic regions of genomes and contain several direct repeats of short sequence with very low sequence variation in consensus frame; repeat chains are dispersed with nonconserved spacer sequences and flanked by about 100-200 base pair leader sequences [45–47]. On the other hand, primary observation showed that the length and number of spacers and repeats in the eukaryotic CRISPR are usually shorter than their prokaryotic homolog. It seems that the length of spacers in a given genome is shorter, and instead, the number of spacers in that genome is higher. This phenomenon may occur due to limited reproductive power, as well as a lower incidence of agents infecting protozoan (viruses, etc.), and consequently, a lower chance of gaining spacers for eukaryotes. Another observed phenomenon that enhances the probability of eukaryotic CRISPR to be functional is the variation in the length of a CRISPR with similar repeats in different populations of a single family. The presence of CRISPR with variable lengths (due to differences in the number and type of spacers), but the similar repeat sequences in some members of a single family in our study, can be indicative for the function of eukaryotic CRISPR. The fact that the organization of these cluster repeats remained conserved in several categories of organism pointed to a more common role of these genomic organizations [45, 48]. For the detection of cas genes, it is necessary to identify open reading frames (ORF) [49]. In the next step, identified ORFs are searched by the MacSyFinder program using the hidden Markov model search of a library of known Cas proteins [50]. The Cas type and subtype are found by analysis of Cas clusters [50–52].

**Figure 2:**
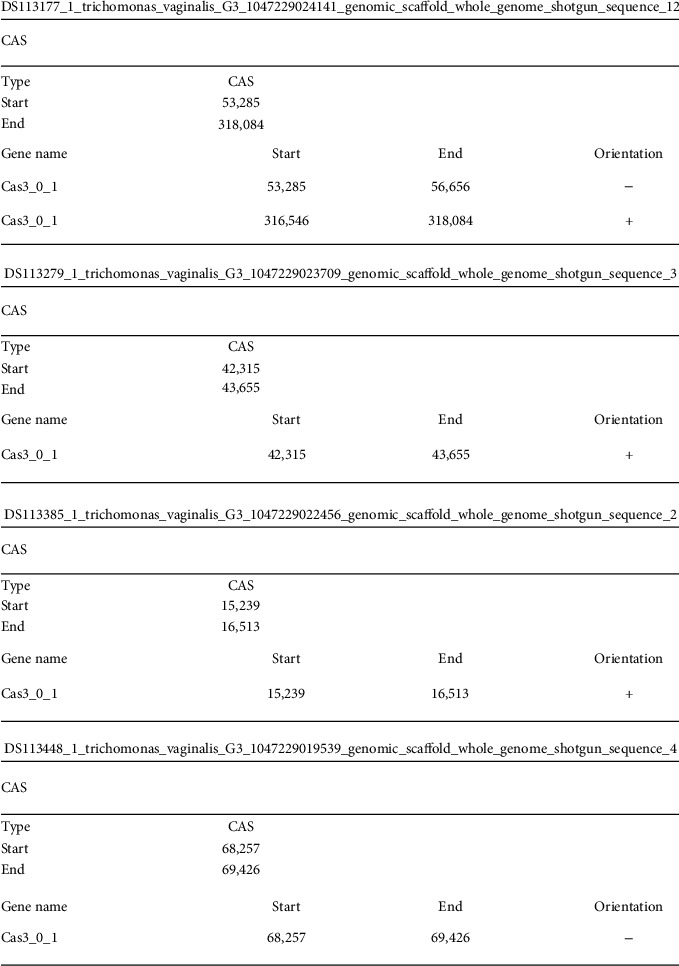
A representative position, common types, and orientation of CAS cluster found in 4 Trichomonas vaginalis isolates.

We found the cas gene of genotype 3(Cas3) in the vicinity of the CRISPR sequence. These genes have a positive orientation in some isolates and a negative orientation in others. Interestingly, in one case, we found two copies of the cas gene in a given isolate having both positive and negative orientation, which may present a functional example of gene duplication. CRISPR's association with the cas gene strongly confirms the organization of the classical CRISPR CAS cluster (Figure 2).

Furthermore, we analyzed some of the bacterial and bacteriophage complete genomic sequences and found the presence of CRISPR sequences in the absence of relative Cas (data not shown), offering different sources for Cas to obtain. On the other side, we found eukaryotic viruses (Orthopoxvirus: Variola virus) that harbour Cas-3 (Cas3_0_I) without having CRISPR sequences and Acanthamoeba mimivirus carrying CRISPR clusters with different evolutionary and evidence levels (data not showed).

## 5. The Origin of New Spacers and Mechanisms Involved in CRISPR Adaptation (Spacer Acquisition)

A similarity search to the intervening sequences was done mainly in the nucleotide sequence database (GenBank-NCBI) using the Basic Local Alignment Tools (BLASTn program) to determine the origin of CRISPR spacer sequences. The preliminary results show that CRISPR spacers have acceptable similarity to original sequences of chromosomal or external transposable genetic elements such as viruses of protozoan parasites (in our primary survey, usually and specially dsDNA viruses, family Mimiviridae), bacteria (mostly Enterobacteriaceae, some lactobacillus, and few other urinary tract-associated bacteria), some transposons, and, interestingly, papillomavirus and HIV-1 in few cases. The CRISPR cluster in Trichomonas seems to acquire new repeat sequences by selective uptake of viral or bacterial DNA, providing promising tools for evolutionary and epidemiologic studies. It is known that intervening sequences of prokaryotic repeats are derived from foreign genetic elements of unrelated organisms. We analyzed the mentioned spacers using BLAST for sequences of the viral genomes and against the complete GenBank database.

Also, even traces of HPV and HIV sequences and other organisms that live in the vaginal area and have a close ecological and epidemiological relationship with Trichomonas were observed, which is confirmed and shown in Table 1 using BLAST.

We detect a series of spacers, some recently acquired by *T. vaginalis* strains, possibly in a clearly polarized fashion. The majority of these spacers belong to the genome of a giant virus. The spacers have homologous parts at different positions in the ancestor genomes.

## 6. Discussion

**Figure 3:**
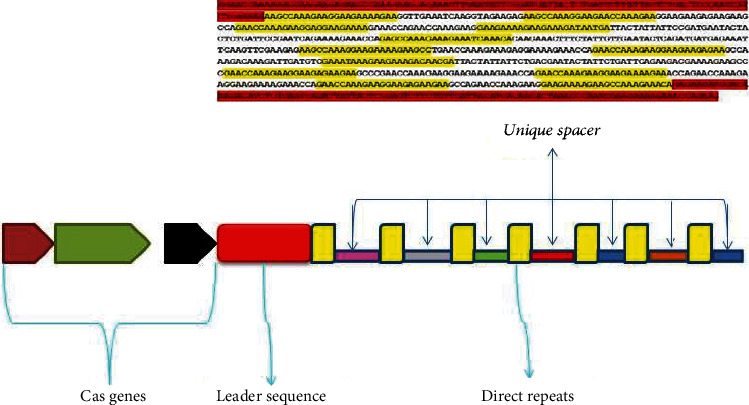
Schematic illustration of a CRISPR Cas system components comprising of a cluster of cas genes, a leader sequence, and repeats and spacers.

Many factors (environmental and host factors or factors related to the pathogen or simultaneous coinfections) can be involved in the pathogenesis of an infection, the host's response and performance, and the disease outcome [4, 6, 8, 16, 23, 25, 33, 34, 36]. The presence and functioning of many of these factors are still unknown. The CRISPR and Cas systems are composed of different adaptation modules and effector components that seem to have at least partially independent evolutionary trajectories (Figure 3). Comparative genome analysis reveals the presence of transposons and transposable elements, Cas protein, homologs of Cas protein, and integrase in our evaluated cells. If our hypothesis is correct and confirmed by experimental laboratory methods, it will not only change our concept of the CRISPR technology but also provide a basis for new approaches to the application of CRISPR in diseases as well. CRISPR sequences, with the widest distribution among repeated sequence family in the world of the genome, were reported in almost all assessed archaeal genomes and about 50 and 1-5% of bacterial and mentioned eukaryotic cells, respectively (-hitherto-). This finding in the first step seems to prove our hypothesis and reject the earlier theory that CRISPR sequences have not been found in any eukaryotic genome to date.

Many of the unusual phenomena and complications that we observe in different microorganisms may be due to systems and mechanisms that are unknown. Given the above, there is a striking similarity between the prokaryotes-Archaeans CRISPR and what we find as eCRISPR. We believe that the unexpected beneficial effects of eCRISPR might be revealed by confirmation of mentioned sequences as functional CRISPR in Eucaryota. Time-dependent increase in repeat and spacer number and length must be shown during the proliferation and life span of carrier cells. CRISPRs size is increased by duplication (replicating) of the repeat sequences and (during) adding a new spacer sequence. The mechanism of spacer addition is still unknown [44]. Only about 5-10% of the 398 sequenced T. vaginalis possess a CRISPR structure. These genomic fragments can act as powerful and easy-to-use phylogenetic markers in complement to other methods. Differences in the detected cluster's length may be that acquiring new spacers is not done at a constant rate in the Trichomonas species life cycle but rather that some unknown conditions can trigger an increased activity. CRISPR sequences and Cas protein genes are present in Trichomonas vaginalis but not in all isolates, as well as the number and type of repeats and spacers, and therefore, the length and sequence composition of these clusters are different in isolates carrying these structures.

Today, in addition to classical studies using animal or microbial models and laboratory experiments, various types of computational models, software, comparative techniques, and predicted three-dimensional structures based on the data obtained from genome-protein sequencing data and computer-aided calculations and designs are developed and used in biomedical research. These methods are capable of predicting and recognizing new genes, proteins, patterns, and biological pathways, or identifying homologous proteins or structures in other types of cells and organisms, or creating evidence or understanding of their presence and function with high precision, accuracy, and cost-effectiveness [28, 31, 36, 39, 45, 48, 50–55].

In the present study, about 50,000 eukaryotic sequences were analyzed, among which there were 398 full genomes of Trichomonas vaginalis. About 10% of Trichomonas vaginalis sequences contained CRISPRK-like structures. In these structures, different combinations of CRISPR and CAS were observed in different sizes and positions.

Based on sequences of putative spacer that we find in this study, it is possible that the T. vaginalis CRISPR CAS system has some role against the attack of genetic elements or virus invading protozoan cells or in horizontal gene transfer probably through a mechanism similar to what occurs in bacteria.

These observations may indicate the presence of a particular function or role for this structure in the host organisms. Comparative analysis showed a high similarity between these structures and prokaryotic crisps in terms of genetic sequencing and organization, suggesting the possibility of acquiring these structures from bacterial ancestors.

**Figure 4:**
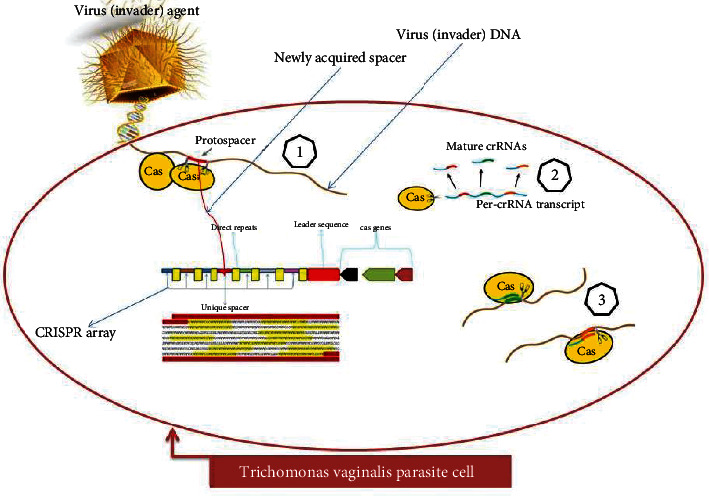
Proposed mechanism of CRISPR/Cas immunity to invader agents (viruses). The CRISPR/Cas system provides immunity to virus, and its main features can be described by three distinct stages: (1) acquisition, (2) processing, and (3) interference.

Lateral gene transfer is a nonsexual mechanism for the transfer (acquisition and fixation) of genetic materials between the recipient organism from a foreign donor cell. It can be proposed as a possible mechanism for a rapid repossession of new capabilities or features like utilization of new metabolites, resistance to or degradation of disinfectants and antibiotics, or defence against invader genetic elements or viruses (bacteriophages in case of bacteria) (Figure 4).

The mechanism for acquisition and uptake of foreign genomes by bacteria like conjugation, transduction, and transformation, or via gene transferring elements such as transposon agents and a more interesting phenomenon, CRISPR CAS systems, are well described. But the mechanisms that are involved in the transfer or acquisition of genes in eukaryotic cells, especially in a natural environment, are not fully known or not well described. Some processes like phagocytosis, transposable element transfers, gene duplications, and LGTs were proposed as possible mechanisms for gene acquisition in Trichomonas vaginalis [6, 8, 23, 24]. Recently, the role of gene transfer (HGT or LGT) has been proposed as an important factor in part of the change in eukaryotic proteome content, and its expansion and evolution, but the mechanisms of acquisition and stabilization of transferred sequences in eukaryotas genome are still unclear. According to previous reported rates and pattern for lateral/horizontal gene transfer in eukaryotic microbes, most of these transferred genes or genetic elements are usually integrated to target genome one-to-one and bordered by genes that are inherited vertically (repetitive sequences) on the chromosome of the host organism (as shown in Figure 1) and are in accordance with common features of classic CRISPR CAS system structures. However, recent findings suggest that transposable genetic elements may also facilitate the acquisition or transfer of genes or genetic sequences from prokaryote or other microbes (like viruses) to unicellular eukaryotes [15, 23, 24, 26, 27, 56].

**Figure 5:**
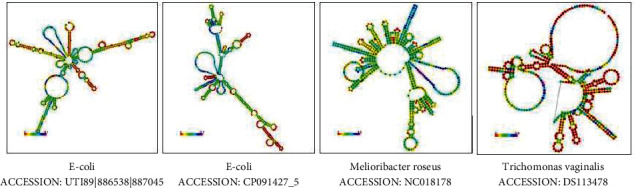
Predicted secondary structure for different CRISPR sequence from different prokaryotic organism and Trichomonas vaginalis. Results have been computed using RNAfold 2.5.1.

Predicted secondary structure for different CRISPR sequence from different prokaryotic organism (two E.coli and Melioribacter roseus) and Trichomonas vaginalis are shown in Figure 5. Results have been computed using RNAfold 2.5.1. webserver (Gruber AR, Lorenz R, Bernhart SH, Neuböck R, Hofacker IL).

### 6.1. The Vienna RNA Website (Nucleic Acids Research, doi:10.1093/nar/gkn188)

The RNAfold service is a web server that can predict the structures of MFE (minimum free energy) and the probabilities of base pair from sequences of single DNA or RNA and will draw secondary structures for single strand DNA or RNA sequences. As can be seen, the difference in the secondary structure between prokaryotes and even between the two CRISPR systems of the two E.coli bacteria and also with Trichomonas vaginalis is observed, which is usually due to the difference in the nucleotide composition of the repetitive sequences and also the difference in the nucleotide sequence of spacers due to the acquisition from different origins.

## 7. Conclusion

According to sequences and their organization, we assume that these repeated sequences and spacer, along with their mentioned features, could be the eukaryotic homolog of prokaryotes and Archaean CRISPR systems and may involve in a process similar to the CRISPR function [45]. Investigation in higher eukaryotes and experimental analysis are recommended. The phenomenon that these unique clusters remained as conserved regions in all domains of cells can show a possible essential and also a more general role for these unique sequences. These cluster fragments were found in approximately all Archean and 50% of bacteria but in less than 5-10% of mentioned eukaryotic cells, making them the widest prevalent family of repeated sequences in the living organism. Here, based on our finding, it seems that it could reject the earlier theory that CRISPR Cas structures and sequences have not been previously described or reported in any eukaryotic genomes [45]. Contrary to classic studies that are primarily based on the frequency of infection and demographic data, newer studies are concentrated on intraspecies molecular characteristics and differences, new phenomena, and unusual complications in a group of organisms, followed by the determination of possible mechanisms and microbe-host interactions along with the frequency of infection and demographic data.

Considering the roles that have been reported for CRISPR CAS systems in bacteria—from drug resistance, pathogenesis, and virulence to the interaction with the human immune system—if the natural presence of CRISPR CAS or structures with similar functions is proven, there will be fundamental changes in our view and practice to the pathogenesis and treatment of many organisms and related diseases.

## Figures and Tables

**Table 1:** Giant viruses found in analyzing spacer using BLAST.

Description	Percent ident by BLAST^∗^	Query cover	Accession number	Family/order
Pithovirus LCPAC101 genomic sequence	88.46%	98%	MK500440.1	Megavirales/Pithoviridae
Klosneuvirus KNV1 Klosneuvirus_5 genomic sequence	91.30%	98%	KY684112.1	Mimiviridae/Klosneuvirinae
Terrestrivirus sp. clone Terrestrivirus_4 genomic sequence	95.45%	100%	MK071982.1	Megavirales: Mimiviridae
Acanthamoeba polyphaga mimivirus isolate M4, complete genome	91.67%	97%	JN036606.1	Megavirales: Mimiviridae
Pithovirus LCPAC001 genomic sequence	89.29%	77%	MK500428.1	Megavirales/Pithoviridae
Mimivirus U306 genome assembly, complete genome: monopartite	86.67%	69%	LT717347.1	Megavirales: Mimiviridae
Acanthamoeba castellanii mimivirus DNA, nearly complete genome, strain: Mimivirus shirakomae	86.67%	74%	AP017645.1	Megavirales: Mimiviridae
Acanthamoeba castellanii mimivirus DNA, nearly complete genome, strain: Mimivirus kasaii	86.67%	74%	AP017644.1	Megavirales: Mimiviridae
Mimivirus Bombay isolate 1 genomic sequence	86.67%	74%	KU761889.1	Mimivirus
Samba virus, complete genome	86.67%	97%	KF959826.2	Samba virus/Mimivirus
Niemeyer virus, partial genome	86.67%	77%	KT599914.1	Mimivirus group A
Mimivirus terra 2 genome	86.67%	100%	KF527228.1	Megavirales: Mimiviridae
Hirudovirus strain Sangsue, complete genome	86.67%	85%	KF493731.1	Mimiviridae/Mimivirus
Acanthamoeba castellanii mamavirus strain Hal-V, complete genome	86.67%	85%	JF801956.1	Megavirales: Mimiviridae
Acanthamoeba polyphaga mimivirus, complete genome	86.67%	100%	HQ336222.2	Megavirales: Mimiviridae
Acanthamoeba polyphaga mimivirus, complete genome	86.67%	64%	AY653733.1	Megavirales: Mimiviridae
Mimivirus sp. SH clone 0003 genomic sequence	91.30%	98%	MH046813.1	Megavirales: Mimiviridae
Pandoravirus neocaledonia, complete genome	100.00%	98%	NC_037666.1	Megavirales/Pandoraviridae
Samba virus, complete genome	86.67%	100%	KF959826.2	Samba virus/Mimivirus
Niemeyer virus, partial genome	86.67%	97%	KT599914.1	Mimivirus group A
Mimivirus terra 2 genome	86.67%	77%	KF527228.1	Megavirales: Mimiviridae
Tupanvirus soda lake, complete genome	91.30%	69%	KY523104.1	Mimiviridae
Uncultured Caudovirales phage clone 3S_14, partial genome	93.10%	74%	MF417874.1	Caudovirales
Tupanvirus deep ocean, partial genome	100.00%	74%	MF405918.1	Mimiviridae
Klosneuvirus KNV1 Klosneuvirus_3 genomic sequence	100.00%	74%	KY684110.1	Mimiviridae/Klosneuvirinae
Pandoravirus dulcis, complete genome	100.00%	70%	NC_021858.1	Megavirales/Pandoraviridae
Pithovirus sibericum isolate P1084-T, complete genome	100.00%	85%	KF740664.1	Megavirales/Pithoviridae
Pandoravirus dulcis, complete genome	100.00%	85%	KC977570.1	Megavirales/Pandoraviridae
Pandoravirus celtis, complete genome	100.00%	85%	MH899943.1	Megavirales/Pandoraviridae
Cedratvirus Zaza IHUMI strain IHUMI-S29 genome assembly, chromosome: scaffold_1	100.00%	85%	LT994652.1	Megavirales
Cedratvirus lausannensis genome assembly, chromosome: cClCRIB-75	100.00%	85%	LT907979.1	Megavirales
Cedratvirus A11 genome assembly, complete genome: monopartite	100.00%	69%	LT671577.1	Megavirales
Harvfovirus sp. clone Harvfovirus_12 genomic sequence	100.00%	85%	MK072254.1	Mimiviridae/Klosneuvirinae
Cedratvirus Zaza IHUMI strain IHUMI-S29 genome assembly, chromosome: scaffold_1	100.00%	69%	LT994652.1	Megavirales
Cedratvirus lausannensis genome assembly, chromosome: cClCRIB-75	100.00%	85%	LT907979.1	Megavirales
Cedratvirus A11 genome assembly, complete genome: monopartite	100.00%	85%	LT671577.1	Megavirales
Pandoravirus dulcis, complete genome	100.00%	85%	NC_021858.1	Megavirales/Pandoraviridae
Brazilian marseillevirus strain BH2014, complete genome	100.00%	94%	KT752522.1	Marseilleviridae
Mollivirus sibericum isolate P1084-T, complete genome	100.00%	85%	KR921745.1	Mollivirus
Pithovirus sibericum isolate P1084-T, complete genome	100.00%	83%	KF740664.1	Megavirales/Pithoviridae
Tunisvirus fontaine2 strain U484, complete genome	100.00%	75%	NC_038511.1	Marseilleviridae/Marseillevirus
Pandoravirus dulcis, complete genome	100.00%	100%	KC977570.1	Megavirales/Pandoraviridae
Pandoravirus celtis, complete genome		77%	MK174290.1	Megavirales/Pandoraviridae
Microviridae sp. isolate ctbi747, complete genome	94.74%	83%	MH617505.2	
Pithovirus LCPAC403 genomic sequence	100.00%	83%	MK500593.1	Megavirales/Pithoviridae
Pithovirus LCPAC304 genomic sequence	100.00%	100%	MK500565.1	Megavirales/Pithoviridae
Hyperionvirus sp. clone Hyperionvirus_7 genomic sequence	100.00%	83%	MK072389.1	Mimiviridae; unclassified Mimiviridae
Hyperionvirus sp. clone Hyperionvirus_4 genomic sequence	100.00%	83%	MK072386.1	Mimiviridae; unclassified Mimiviridae
Terrestrivirus sp. clone Terrestrivirus_4 genomic sequence	100.00%	83%	MK071982.1	Mimiviridae; unclassified Mimiviridae
Pithovirus LCPAC302 genomic sequence	100.00%	64%	MK500544.1	Megavirales/Pithoviridae
Pythium polare RNA virus 1 PpRV1-OPU1176 genomic RNA, complete genome	100.00%	98%	NC_040608.1	Totiviridae
Cedratvirus Zaza IHUMI strain IHUMI-S29 genome assembly, chromosome: scaffold_1	100.00%	98%	LT994652.1	Megavirales
Cedratvirus A11 genome assembly, complete genome:	100.00%	85%	LT671577.1	Megavirales
Barrevirus sp. clone Barrevirus_16 genomic sequence	100.00%	97%	MK072013.1	Mimiviridae; unclassified Mimiviridae
Barrevirus sp. clone Barrevirus_3 genomic sequence	100.00%	97%	MK072000.1	Mimiviridae; unclassified Mimiviridae
Satyrvirus sp. clone Satyrvirus_31 genomic sequence	100.00%	97%	MK072467.1	Mimiviridae; unclassified Mimiviridae
Terrestrivirus sp. clone Terrestrivirus_5 genomic sequence	100.00%	97%	MK071983.1	Mimiviridae; unclassified Mimiviridae
Acanthamoeba polyphaga moumouvirus, complete genome	95.65%	69%	JX962719.1	Mimivirus/moumouvirus
Moumouvirus Monve isolate Mv13-mv, partial genome	95.65%	69%	JN885998.1	Mimivirus/moumouvirus
Megavirus lba isolate LBA111, complete genome	83.33%	69%	JX885207.1	Megaviridae
Megavirus chiliensis, complete genome	83.33%	74%	JN258408.1	Megaviridae
Mimivirus sp. SH clone 0001 genomic sequence	95.24%	74%	MH046811.1	Megavirales: Mimiviridae
Circular genetic element sp. isolate ctcf325, complete sequence	82.35%	72%	MH618190.1	
Powai lake megavirus isolate 1, complete genome	92.00%	70%	KU877344.1	Megaviridae
Mollivirus sibericum isolate P1084-T, complete genome	91.30%	45%	KR921745.1	Mollivirus
Hepelivirus isolate HepeV ORF1 gene, partial cds; and ORF2 gene, complete cds	100.00%	85%	JQ898340.1	Unclassified RNA viruses
Dasosvirus sp. clone Dasosvirus_10 genomic sequence	100.00%	100%	MK072051	Mimiviridae; unclassified Mimiviridae
Tokyovirus A1 DNA, nearly complete genome	100.00%	100%	AP017398.1	Marseilleviridae; Marseillevirus; unclassified Marseillevirus
Pandoravirus dulcis, complete genome	95.24%	100%	KC977570.1	Megavirales/Pandoraviridae
IAS virus, complete genome	100.00%	100%	KJ003983.1	Unclassified bacterial viruses; crAss-like viruses
Pithovirus LCPAC401 genomic sequence	100.00%	100%	MK500585.1	Megavirales/Pithoviridae
Bos taurus papillomavirus 17, complete genome	100.00%	100%	KU519392.1	
Acanthamoeba castellanii mamavirus strain Hal-V, complete genome	100.00%	100%	JF801956.1	Mimivirus/mamavirus
Pithovirus LCPAC406 genomic sequence	100.00%	50%	MK500607.1	Megavirales/Pithoviridae
Hubei partiti-like virus 7 strain QTM23269 RdRp gene, complete cds	100.00%	81%	KX884117.1	Riboviria; unclassified RNA viruses
Hirudovirus strain Sangsue, complete genome	100.00%	76%	FJ373894.1	Mimiviridae/Mimivirus
Pithovirus LCPAC104 genomic sequence	88.46%	72%	MK310184.1	Megavirales/Pithoviridae
Klosneuvirus KNV1 Klosneuvirus_1 genomic sequence	95.24%	63%	KY684108.1	Mimiviridae/Klosneuvirinae
Megavirus vitis isolate vigne, complete genome	92.31%	49%	MG807319.1	Megavirus
Bandra megavirus isolate KK-1 genomic sequence	92.31%	64%	MG779390.1	Unclassified Mimiviridae
Megavirus terra1 genome	92.31%	98%	KF527229.1	Megaviridae
Megavirus lba isolate LBA111, complete genome	92.31%	98%	JX885207.1	Megaviridae
Megavirus courdo11, complete genome	92.31%	77%	JX975216.1	Megaviridae /Mimivirus group C
Megavirus courdo7 isolate Mv13-c7, partial genome	92.31%	83%	JN885993.1	Megaviridae/Mimivirus group C
Megavirus chiliensis, complete genome	92.31%	75%	JN258408.1	Megaviridae

^∗^ stands for Basic Local Alignment Search Tool.

## Data Availability

The readers can access the data supporting the conclusions of the study by rational request to the corresponding author.
